# Nurses' experiences and perspectives regarding evidence‐based practice implementation in healthcare context: A qualitative study

**DOI:** 10.1002/nop2.2080

**Published:** 2024-01-15

**Authors:** Rasha A. Mohamed, Muhanad Alhujaily, Faransa A. Ahmed, Wael G. Nouh, Abeer A. Almowafy

**Affiliations:** ^1^ Department of Nursing, College of Applied Medical Sciences University of Bisha Bisha Saudi Arabia; ^2^ Department of Medical Laboratory Sciences, College of Applied Medical Sciences University of Bisha Bisha Saudi Arabia; ^3^ Department of Nursing, College of Applied Medical Sciences in Alnamas University of Bisha Bisha Saudi Arabia; ^4^ International Islamic Center for Population Studies and Research Al‐Azhar University Cairo Egypt

**Keywords:** clinical practice, evidence‐based practice, experiences, healthcare, implementation, nurses, perspectives

## Abstract

**Aim:**

To explore nurses' experiences and perspectives about evidence‐based practice (EBP) implementation in the healthcare context.

**Design:**

A qualitative descriptive study design using focus group discussions (FGDs).

**Method:**

Sixty‐four nurses who purposefully selected and worked at various healthcare organizations in Bisha Governorate, Saudi Arabia, were included. Eight FGDs were used to obtain data using open‐ended questions. The collected data underwent inductive qualitative content analysis.

**Results:**

Two main categories were extracted: experiences and perspectives towards EBP. The experiences category emerged into four sub‐categories: familiarity with concepts and benefits, steps, dissemination sources of EBP and sources of EBP knowledge, while perspectives towards the EBP category included four sub‐categories: application of EBP in clinical practice, barriers, facilitators and EBP application methods. The participants experienced being in a non‐supportive and non‐encouraging atmosphere which results from a lack of organizational commitment to EBP implementation and illuminates the complexities involved in the integration process.

**Conclusion:**

The nurses' experience with EBP indicated that there was limited support for the implementation of this approach. Furthermore, they experienced varying barriers to EBP implementation. They viewed EBP as a complex technique as they lacked knowledge and skills related to the formulation of research questions, and retrieving, applying and disseminating of EBP in clinical practice decision‐making. That is due to barriers pertinent to the individual, organizational and patient factors. The key to successfully implementing an EBP in nursing practice is to promote professional development, comprehensive and continuous training, a culture of change, organizational support and motivation.

**Clinical Relevance:**

Through the construction and provision of ongoing educational interventions and mentoring programmes about EBP, healthcare organizations and nursing leadership may develop a comprehensive strategy to encourage staff nurses' participation in the EBP process. This is to enhance nurses' experiences and perspectives towards the EBP approach and overcome the barriers to effective implementation.

**No Patient or Public Contribution to this Study:**

Patients or the general public were not involved in the design, analysis or interpretation of the data in this study.

## 1 INTRODUCTION

Evidence‐based application of nursing practice has become a significant matter in clinical nursing practice and the healthcare system since it is considered the gold standard for delivering high‐quality, safe and affordable healthcare globally (Alqahtani et al., 2020; Kumah et al., 2022). EBP refers to the application or translation of research findings to the routine administration of patient care and clinical procedures (Portney, 2020). EBP offers several benefits, including enhancing the nurses' leadership abilities, expanding their critical thinking skills and enhancing their capacity to serve patients with safe and valid practices (Brunt & Morris, 2023; Ramage & Foran, 2023). It also improves the standard of healthcare services and cost management in the framework of the healthcare system, lowering the risk of medical error and patient mortality (Dang et al., 2021). Furthermore, EBP encourages professional development and lowers diversity in clinical practices, thus decreasing mortality and fostering patient satisfaction (Chovanec et al., 2021).

One of the main health professions that can greatly impact how well healthcare is provided is nursing (Pierce, 2020). High‐quality nursing care is urgently needed in the current healthcare environment, and this demand is spreading globally. Evidence‐based practice in nursing involves providing holistic, quality care based on the most up‐to‐date research and knowledge rather than traditional methods, advice from colleagues or personal beliefs (Chan et al., 2023). Thanks to their abilities in leadership, teaching, teamwork, communication, evaluation, providing feedback and gathering and modifying the best available information, nurses are among the clinicians who are most important in putting evidence into practice and enacting long‐lasting changes (Ten Ham‐Baloyi, 2022).

In Saudi Arabia, there has been a significant change in the nursing workforce from a small number of nurses with basic nursing skills to qualified nurses with advanced nursing practice abilities, which created a problem in clinical settings. Due to the difficulty in providing high‐quality healthcare, the Saudi government created Vision 2030 (National Transformation Program), most of which can be achieved by implementing EBP. The challenge is that Saudi nurses are accustomed to traditional, un‐evidenced caregiving methods (Alshehry et al., 2019). To provide high‐quality nursing care, governmental and private organizations in the Kingdom of Saudi Arabia (KSA) recommend enhancing evidence‐based competencies for nurses. The KSA has recently focused more on establishing a modern, reliable healthcare system that provides services with a quality guarantee (Ma'moun & Abu‐Moghli, 2020).

It can be seen in the literature that nurses do not have complete knowledge and competence in the context of EBP. The ability to formulate questions at work, analyse them, critically evaluate the information and apply the results to the patient care process demands a broad range of abilities. Nursing practice is still not evidence based, despite the availability of novel research‐based knowledge and published articles with the potential to improve nursing care quality and advance EBP (Michelle & Troseth, 2022). Also, developing skills in this area is a multi‐faceted activity that is revealed in the discrepancies between ‘what the evidence says’ and ‘what is done in clinical practice’ (Loura et al., 2021).

EBP implementation and quality improvement (QI) were identified as essential skills that all healthcare professionals (HCPs), particularly nurses, should possess as front‐line healthcare workers. It is essential to assess the nurses' current level of knowledge, abilities and attitude towards EBP and QI to improve the quality of patient care, increase nurses' competencies in such practices and significantly influence the improvement of healthcare quality (Hashish et al., 2020).

In nursing, investigating the factors that can provide nurses and decision‐makers with more insight into the implementation and difficulties related to adopting and implementing EBP is mandatory (Hasanpoor et al., 2019). Although quantitative research has investigated nurses' attitudes and beliefs towards EBP, not many qualitative studies have been found in the Saudi Arabian literature. Thus, this study aimed to explore the nurses' experiences and perspectives towards EBP implementation in a healthcare context to fill this knowledge gap. Future research that concentrates on applying the EBP will build on the findings of this study. The study results are meant to add to the body of knowledge and aid in developing new projects and initiatives in this field.

### 1.1 Aim of the study

To explore nurses' experiences and perspectives towards EBP implementation in the healthcare context, this study explored the following questions:

## 2 METHOD

### 2.1 Study design and setting

A qualitative descriptive study design was employed to explore and provide deep insights into the experiences and perspectives of the nurses towards EBP implementation in the healthcare context. It was believed that the qualitative method would be an appropriate method of identifying in depth what nurses know and think concerning EBP (LoBiondo‐Wood & Haber, 2021). This study was carried out at various healthcare organizations (HCOs): two governmental hospitals, namely King Abd Allah Hospital and Labor and Children Hospital; and four primary healthcare centres (PHCs) (Bisha, Nemran, Mattar and Ganoub El Madina), Bisha Governorate, Saudi Arabia, from November 2022 to February 2023. The hospitals provide various healthcare services, from emergency to rehabilitation, including in‐patient and out‐patient services. The primary healthcare centres provide maternal and child healthcare services, and communicable and chronic disease prevention and control.

### 2.2 Participants

In this study, the inclusion criteria were as follows: nurses currently working in different departments at the selected hospitals and healthcare centres for at least 1 year, having at least a bachelor's degree in nursing sciences, being willing to participate in the study, being willing to share their work experiences and signing the informed consent form, while nurses with less than 6 months of experience and interns were excluded. A total of 64 participants were recruited via purposive sampling to ensure informants were relevant for the study (until saturation was reached and a point of informational redundancy was reached). As per the literature (Akyıldız & Ahmed, 2021), it was decided that the appropriate number of individuals to participate in the focus group discussions (FGDs) would be six to eight. Moreover, the chosen participants must have enough information to debate the subject of the discussion. Towards this aim, the selection of the group participants was carried out with the method of ‘homogeneous sampling’, one of the techniques of purposive sampling and through contact with the selected healthcare organizations' managers to coordinate the meeting with the nurses. Four FGDs were formed at the two hospitals; each focus group was composed of 10 participants. While at the PHC, four FGDs were formed and included six participants each. A variety of ages, both sexes, different educational levels and uneven years of clinical experience in the nursing field among the participants were emphasized. The majority of participants worked in direct care of patients, assessing patients' needs and making decisions on nursing interventions.

### 2.3 Data collection

An official letter from the nursing department of the College of Applied Medical Sciences was submitted to the administrative authorities in the selected healthcare settings for obtaining authorization and support during the data collection period. The participants were recruited through verbal invitations and with the facilitation of the nurse managers.

A list of open‐ended questions and follow‐up questions was prepared as guidance for each FGD based on the research's aim to clarify and explore the similarities and differences in experiences and perspectives. The study questions for the FGDs were adopted from a previous study done by Elsayd et al. (2019). Permission to use the questions was obtained from the copyright holder. The questions were translated from English into Arabic and vice versa to ensure meaning consistency and to suit the participants' cultures. Furthermore, before conducting the study, the Arabic version of these questions was piloted on seven nurses (who were not included in the study sample) to assess the study tools' clarity, application and reliability. In addition, to determine the amount of time required for data collection. The questions were changed (rewording a few statements) based on the pilot test results.

Examples of the questions included: (a) Experiences about EBP implementation in healthcare: What is the concept or approach of EBP? What are the benefits of EBP? What are the steps of EBP? What are the scientific databases and search engines used in EBP? What are the sources of knowledge that inform clinical practice decision‐making? From your experience, what are the methods of EBP dissemination? What are your sources of knowledge related to EBP?; (b) Perspectives towards EBP implementation in healthcare: What are your perspectives on the effect of applying EBP in nursing practice? What are the barriers that hinder the application of the EBP approach? What are the required facilities for applying the EBP approach? From your experience, what are the most applicable methods for applying EBP at HCOs? The personal and occupational data of the nurses, such as age, sex, educational qualification, work settings, years of experience and previous exposure to any educational training related to EBP, were also completed.

FGDs were held in a private and quiet room in the selected healthcare settings to minimize distractions and interruptions and according to nurses' schedules to ensure participants' comfort. Before the FGDs began, the ground rules were established, and good communication flowed from the greeting. The researchers urged the participants to respect each other's confidential information, not to share information outside the group and not to reveal the identities of any of the other members of the focus group to anyone.

Four researchers took part in the data collection in a distinct constellation. The first author (R. M.) moderated all FGDs, and three of the authors (F. A., W. N. and M. A.) co‐moderated. Additionally, the co‐moderators wrote field notes during the FGDs, probed for clarification and, after the discussion, provided a summary of the conversation for the participants to revise or elaborate on. We performed eight FGDs lasting approximately 30–45 min each due to the work nature of the nurses.

The discussions began with the participants introducing themselves to one another to enhance the sense of rapport. The researchers made sure that every participant spoke about their feelings, concerns and worries. The participants are actively encouraged to not only express their own opinions but also respond to other members and questions posed by the moderator. What is more, the researchers tried to create a non‐intimidating environment so that participants felt free to talk openly and give honest opinions.

During FGDs, the researchers tried to avoid using negative, forgiving or judging remarks and attitudes. The researchers used questions at the end of the FGDs, such as ‘Anything else?’ or ‘Do I need to know anything other than what I have asked you?’. When there were several overlapping replies from different participants and the researchers heard the same responses come up again and again with nothing new, saturation was achieved after six FGDs in the themes of the benefits of EBP and the barriers to EBP. The data from the FGDs were recorded, and then the audio was transcribed verbatim (word by word) and anonymized directly during transcription. For a better understanding of the context and a more concise interpretation of the discussions, field notes (themes, hunches and ideas) were used for data analysis. These notes capture non‐verbal information (body language and expression) and serve as a backup. Data were collected over 2 months.

### 2.4 Research rigour

The rigorous criteria, including credibility, transferability, dependability and confirmability, were addressed to ensure the reliability of this study (Lindgren et al., 2020). Credibility was achieved through member checks. The researchers returned the results to the participants during the member check to ensure they were accurate and in line with their experiences. It was also accomplished by providing a comprehensive explanation of the research methods and processes. Quotations are used in the result section to illustrate the findings. The quotations were translated from Arabic to English by the two researchers (R. M. and M. A.). The manuscript proofreader, who had access to both the translated and original quotations, reviewed the translations for correctness in English. By clearly describing the study's background and confirming that the data were representative, transferability was ensured. By carefully selecting a variety of FGD participants who provided a wealth of comprehensive data to detect saturation levels, representativeness was achieved. For additional reviewers to confirm the results, a transparent and comprehensive description of the processes has been provided. Through precise and in‐depth documentation of the developed categories and sub‐categories, confirmability was achieved.

Additionally, the utilization of research triangulation in the analysis process improved the reliability of the results. Two of the authors are assistant professors in the nursing field, one is a senior professor and one is an associate professor in medical laboratory sciences, while one researcher is a Ph.D. holder with a medical specialist in gynaecology. Among us, three are female. Two of the researchers (R. M. and A. A.) had previous experience in qualitative research. All researchers had extensive experience in teaching and research studies. Each researcher noted their prior knowledge before the data were gathered and analysed to raise awareness of it, make deliberate use of it and encourage a deeper understanding of what was being studied. Furthermore, the researchers received training in qualitative research design, discussion and analysis. To ensure that personal characteristics do not influence the research process, the researchers maintained a reflective stance by bracketing and putting aside personal biases, assumptions, beliefs and experiences.

In addition to providing enough time for data collection and analysis, the researchers engaged participants for extended periods, maintained polite and acceptable communication and regularly discussed the retrieved codes with several participants. They stated that the themes reflected their experiences. Moreover, developing these study questions was done by asking ‘how and why questions’, meaning the questions formulated are exploratory and aim clearly to identify what is known about a phenomenon from one or more perspectives with consideration of the settings and environmental context. Congruence among the research aim, questions, research design, data collection and analysis to address the question was emphasized during this study implementation. Besides, each finding that was extracted was supported by an illustration that is a verbatim extraction of the words of the participants.

### 2.5 Data analysis

The data were illustrated by the conventional content analysis method presented by Lindgren et al. (2020) through an inductive coding approach for a more expansive analysis of the entire body of data. Conventional content analysis is a powerful yet flexible method for analysing qualitative data that can be used to understand experiences, thoughts or behaviours across a dataset for developing codes, categories and themes from textual data instead of from pre‐existing theories (Kiger & Varpio, 2020).

To ensure the validity of the content analysis, the three authors (R. M., M. A. and A. A.) first went through all of the field notes and discussion transcripts. After that, R. M. and A. A., two of these authors, separately and painstakingly extracted and coded meaning units associated with the aim, discussing their differences and disagreements with the author (M. A.) until an agreement was reached. Following a comparison of the coded meaning units for similarities and differences, codes with related content were categorized into subthemes. Eventually, themes were formed by grouping‐related subthemes. The process of analysis was iterated until all the researchers concurred that all text about the aim had been incorporated and categorized according to an appropriate theme. The common themes were also provided to two qualitative research experts who were not participating in the study for verification. Finally, they verified and identified the common themes. Two main categories were extracted: experiences and perspectives towards EBP.

### 2.6 Ethical considerations

The Declaration of Helsinki, first published in 1964, and subsequent revisions set forth the ethical guidelines that were used in performing this study. The study protocol was approved by the University of Bisha's Institutional Review Board (UBCOM‐RELOC) (H‐06‐BH‐087/(03.01.23)). All research participants verbally concurred after a comprehensive information presentation. The participants were also informed that they might withdraw from the research at any time and for any reason. The study's data would be kept confidential, used only for research and assessed without disclosing the identity of the participants. During the data analysis, the participants' names were coded as numbers. With concerns about risk, there was not any risk linked to participation. According to the guidelines of scientific research, the gathered and analysed data were kept and stored in secured files. The study findings were shared with the participants for their perspectives and feedback on the appropriateness of the themes. The results were published openly and honestly.

## 3 RESULTS

The mean age of participants was 30.31 ± 4.61 years; 22 male and 42 female participants; 42 participants had a bachelor's degree in nursing sciences, 13 had a post‐graduate degree and 9 participants had a diploma in nursing. Forty participants are working in hospitals and 24 in healthcare centres. Regarding work experience; 9 participants had work experience of <5 years, 15 participants worked from 5 to <10 years, 34 participants worked 10 to <15 years and 6 participants worked from 15 to 20 years. Forty‐eight participants did not attend any training programmes related to EBP.

After analysing the data, the main study findings were the categories of experiences and perspectives towards EBP. Both the experiences and perspectives towards EBP categories included four sub‐categories. The sub‐categories have been described below using direct quotations from the participants.

### 3.1 Participants' experiences of the EBP approach

#### 3.1.1 Familiarity with EBP concept/approach and benefits

While respondents were familiar with the term ‘evidence‐based practice’, interpretations of the definitions of EBP varied considerably. In‐depth understanding of EBP notably varied among the nurses. The participants indicated that joining scientific discoveries and clinical trials in clinical practice is the familiar concept of the EBP approach. They viewed it as a method of clinical practice that adheres to scientific methodology and is governed by rules. They considered EBP as a clinical practice guideline or standard of care for providing high‐quality patient care with minimum cost and less effort.

The main thing that crossed my mind was that EBP is how one performs the clinical practice based on the results of experiments, guidelines, or clinical trials. (FGD 1, P2)

I think EBP has not yet been put into practice. A few years ago, while I was employed at another hospital, I became aware of the concept of EBP. My knowledge of EBP is limited. (FGD 6, P3)

Three benefits of EBP for patients, HCPs and healthcare systems were stated by the participants.

- Benefits for patients

The participants expressed that EBP helps improve disease progress and reach patients' satisfaction related to healthcare. Several participants shared similar perceptions that EBP can prevent medical‐related errors and harms, and decrease patients' hospital stay. Additionally, EBP entrusts the patient with a professional commitment.

When we adopt EBP … disease progression will be improved and our patients will be satisfied with the provided care. (FGD 2, P4)

In the ward, we've had a lot of patients. When we use EBP, it will aid in decreasing patients' length of stay at our hospitals, anticipating care‐related mistakes, and preventing errors. (FGD 6, P5)

b Benefits for the healthcare professionals

As the nurse gains experience in applying EBP, it will be applicable to access to logical, scientific and updated knowledge and professional skills. In expansion, EBP makes strides in clinical practice and gives more self‐esteem and confidence. The participants saw the positive outcome when relying on data for research and clinical practice, and how EBP reduces uncertainty and keeps consistency among healthcare professionals. Furthermore, EBP brings about an awareness of what it means to be a nurse and what that nurse's professional responsibility is.

We have to say, that EBP will give us valid, reliable, and recent scientific knowledge and skills. Not just to find an answer, but to find the right answer for the treatment. (FGD 3, P1)

I would love to base my work on evidence, I would be happy if I could investigate at work…. As a nurse, EBP provides me with a variety of opportunities and EBP allows me to manage my practice better. (FGD 7, P5)

c Benefits for the healthcare systems

The participants asserted that when HCOs implement EBP methods, it will help in reducing the cost spent on ineffective treatment. They claimed that the adoption of the EBP approach provides high‐quality healthcare, increases the organizational reputation and gives legal backing. They believed that it makes the profession more valuable and reinforces it.

In fact, in our clinical practices, applying EBP techniques will enhance the professional image and provide the best cost‐effective healthcare. (FGD 7, P2)

When we all use EBP and work in the same way, our work will be organized, safe, and of good quality. (FGD 1, P4)

#### 3.1.2 Steps of EBP

EBP steps were hardly mentioned by the participants. However, they communicated EBP steps as the research proposal steps. On the other side, most of the participants had no information concerning the different types of research questions. Nevertheless, they mentioned only one type of clinical question: the intervention question. They believed that the majority of research studies done are descriptive in nature. Moreover, the majority used Google as their primary source of information. It is worthy of notice that the use of electronic information sources (medical journals and databases) such as PubMed and Medline among nurses was the least common.

From my experiences as I made before a thesis, evidence is the same as a research study. (FGD 7, P4)

I only work with the knowledge that I have gained from my education, and sometimes I use Google to update my practice records. I frequently used Google searching as a quick source of information. (FGD 5, P1)

#### 3.1.3 Dissemination sources of EBP

About the way in which the knowledge and the associated evidence‐based interventions are shared on a wide scale, a variety of channels is present as stated by the participants. Adoption of the clinical practice guidelines and the targeted distribution of information and intervention materials to the clinical practice personnel, publishing research findings in scientific research journals, holding educational training programmes and use of online scientific websites were considered sources for EBP dissemination. Regarding how new evidence is disseminated, most participants concurred that they had official work hours during which they attended educational sessions held by subject‐matter experts.

Of course, to distribute research results, publishing in scientific journals is the way. (FGD 6, P5)

It's better to disseminate new knowledge, particularly through attending periodic educational seminars and meetings, training programs, and workshops especially, in the nursing field. (FGD 2, P7)

#### 3.1.4 Sources of knowledge about EBP

The participants mentioned that the educational seminars and conferences, experience from colleagues as peers, head nurses and clinicians, social media applications (apps) and the internet web were steadily reported as the main sources for retrieving information to make clinical practice decisions. The participants thought the data they obtained from social networking apps were accurate, dependable and trustworthy. However, they lacked awareness of the Cochrane collaboration as an essential source of knowledge about EBP.

If we have time, we prefer to attend training workshops and scientific conferences. We can share information in these sessions. (FGD 3, P7)

Usually, the quickest thing is to ask a colleague. (FGD 2, P6)

### 3.2 Nurses' perspectives towards EBP approach

This theme indicates that the participants experienced a non‐supportive and non‐encouraging atmosphere towards EBP implementation in healthcare. Facilitation was formed through receiving integrated and collaborative training, resource allocation and motivation.

A learning organization, ongoing training, transparent leadership and access to the best available evidence in the form of clinical guidelines or protocols with patient partnership are all essential as crucial components linked to evidence adoption.

### 3.3 EBP application in clinical practice

According to the nurses, EBP is regarded as a beneficial and multi‐faceted approach. Organizational changes should be emphasized. However, applying EBP is a difficult task that calls for highly skilled personnel and encouraging and supportive healthcare environments. Furthermore, they emphasized that the knowledge and clinical procedures will evolve as EBP is applied.

So I would say that… when we apply EBP, we will face many challenges as it requires highly trained human staff and various financial and technical resources. This is fundamental. (FGD 1, P3)

Here, our healthcare organization should encourage EBP implementation and support the culture of change. EBP calls for changes in all organizational systems. (FGD 7, P6)

### 3.4 EBP barriers to EBP application

Barriers to the implementation of EBP emerged more frequently than advantages from its application. The participants classified barriers to EBP application into individual, patient factors and organizational. Doubts about new evidence and resistance to change were the most frequently mentioned obstacles. The participants' preference for traditional care, lack of readable and understandable research evidence and lack of knowledge and skills of EBP were reported by the nurses. A lack of information technology skills, access to research articles and a lack of references in the native language – because most research is published in a foreign language – were all individual factors to EBP application. Furthermore, they were unable to distinguish between high‐quality research studies.

I wish we could practice EBP very well, it will be a bit difficult right now to fully practice EBP. Significantly, the evidence may work for some patients, but may not work for patients in Saudi Arabia. We preferred to practice in ways we had learned in nursing college or that were consistent with routines. (FGD 6, P2)

Unfortunately, as a nurse, I am unable to locate, evaluate, or apply evidence because of my limited expertise in scientific research. Because the bulk of nursing research journals were published in English, I believe that EBP was hindered by the absence of legible and intelligible research evidence. (FGD 1, 7, P4, 3)

The FGDs revealed that the participants noted that the main organizational barrier is the way of management due to leaders' lack of understanding and commitment to the implementation of EBP. Besides, a non‐supportive environment, a lack of time, a heavy workload related to the nature of the nursing profession, overcrowding, a deficiency of workforce, a lack of change culture in the HCOs and a lack of EBP training and education. The participants concurred that although the EBP has theoretical backing, it is very challenging to alter established norms. Some felt that guidelines, when they were available, lacked sufficient information about how to be applied in real‐world situations.

We are understaffed. I am very frustrated; my hospital does not support us to apply EBP because of lack of time and heavy workload. To be clear, we merely wish to complete the tasks required by the leaders, and ensure there are no medical errors. (FGD 1, 3, P4, 1)

I am not motivated to apply new approaches because most scientific databases are not accessible to me. I usually use my bundle on my phone, but it is expensive to download documents to read. (FGD 7, P5)

The participants described patients' barriers to EBP application as patients' health conditions and their concerns related to the cost of care and communication barriers. Also, unfamiliarity with the novel treatment, when patients did not turn out the way they wanted, nurses said they were afraid they would be blamed or verbally and physically abused and, in many instances, they do not need renowned for getting care from a new or unfamiliar nurse. As a result, they were afraid to consider doing anything that might be seen as outside of standard procedures, novel or costly practice compared to the norm, especially the elderly and their families due to the generational culture gap.

We tend to be cautious here; we won't employ new medications or nursing techniques until they are widely recognized because if there is a negative outcome, the patient will be upset and blame us. (FGD 3, P7)

Patients deny applying any new trial. Their families are concerned about the cost. For instance, the evidence suggests that we might use a superior substance to aid patients' recovery, but the patients could not afford it. (FGD 1, P8)

### 3.5 EBP facilitators for application

Of course, the healthcare organization should provide free internet access for us to participate in research activities. Also, providing free time without clinical assignments. (FGD 7, P6)

I think, we need a periodic EBP training program in a rotating method that covers all health workforces. (FGD 1, P3)

By employing a qualified staff, we can use EBP. The healthcare organization should recruit more HCPs to alleviate the work burden and with prior knowledge and skills in EBP. (FGD 6, 8, P1, 2)

The participants highlighted various facilitators that may facilitate EBP adoption as effective allocation, provision of the needed resources and time for EBP implementation. Furthermore, the hiring of knowledgeable and skilled staff and continuous staff capacity building with internal and external collaboration should be done by the healthcare organization. They think nursing administration needs to be in charge of inspiring, motivating and generating nurses' interests and encouragement should come after training.

### 3.6 EBP application methods

According to the participants, the educational interventions, adopting clinical practice guidelines and clinical protocol were the most typical strategies for implementing the EBP approach. EBP incorporates the use of literature, research findings and professional knowledge in healthcare with patient partnership, especially when making clinical decisions. The participants emphasized that they followed routinely the Ministry of Health's recommendations as the clinical protocol for infection prevention and control.

Actually, my hospital provides us with many printed educational materials as clinical guidelines to inform us about new practices or evidence and we may apply them in our clinical practices to overcome the problems in producing national guidelines. I think it is easy to use, and applicable in our clinical practices. (FGD 7, P2)

Personally, I think that implementation of EBP can be done by using of research findings, guidelines, and hospital protocols with patients' partnership in decision‐making. (FGD 2, P6)

## 4 DISCUSSION

Evidence‐based practice competencies are important competencies that nurses must possess. It is necessary to obtain useful scientific information carefully and discriminately to guide decision‐making. Nurses with strong EBP ability can quickly select the strongest evidence from existing information to formulate new strategies and measures when faced with new things, to facilitate a faster and more scientific response to public health events (Zhou et al., 2022). However, the transfer of knowledge into care practice by nurses is progressing slowly due to several barriers: lack of knowledge of EBP and EBP competence, misconceptions or negative attitudes towards research and evidence‐based care, lack of time and resources and organizational constraints, such as lack of support and incentives (Melnyk & Raderstorf, 2019).

EBP is a novel method in Saudi Arabian society; this qualitative study explored nurses' experiences and perspectives about EBP implementation in the healthcare context. According to the findings of this current study, the experiences of nurses indicated that there was limited support for the implementation of this approach. In addition, they experienced negative perspectives of EBP because of barriers related to organizational, individual and patient factors. The possible explanation is attributed to a lack of access to scientific and information resources and a lack of motivation that prevent nurses from acknowledging the EBP approach. Studies done by Elsayd et al. (2019) and Valizadeh et al. (2020) found the same findings. Furthermore, these findings agree with a study by Zammar (2022), which concluded that most nurses are uninformed of the significance of EBP in their profession. However, these findings contradict a study by Kaseka and Mbakaya (2022), who concluded that the nurses viewed EBP positively, and their attitude towards EBP seemed more positive. However, they did not use it much because they lacked the organizational support and competence to do so.

Regarding nurses' experiences of EBP, the current study found that the participants are hardly familiar with the definition of EBP. This finding is comparable to a study by Alshehri et al. (2017). In contradiction, nurses with access to scientific resources and EBP training programmes, on the other hand, were aware of this concept (Claudino et al., 2019). The participants of this study declared that EBP results in favourable outcomes, safe patient care, less time spent on nursing operations and lower treatment costs. The same was found by Dang et al. (2021).

Regarding nurses' experiences of EBP steps, few participants properly mentioned all the steps of EBP, according to the current study. This finding is consistent with previous research, demonstrating that most HCPs were unaware of EBP steps (Tolera & Feng, 2017). However, this finding is in contrast to a study in which the participants participated in research activities and practised EBP (Lafuente‐Lafuente et al., 2019).

Regarding nurses' experiences with search engines and scientific databases, it was discovered that the participants used Google as their primary search engine. This is owing to the other scientific databases' lack of knowledge and skills. This finding is in line with the study's findings by Zigdon et al. (2020), which revealed that the participants used Google as a common source of information. Furthermore, according to this study, some nurses do search by using PubMed. This result is consistent with the findings of Lafuente‐Lafuente et al. (2019), who found that participants were aware of databases and searching skills and participated in research activities.

In terms of nurses' perspectives towards the EBP approach, the outcomes of this study revealed that the participants asserted the benefits of EBP. EBP is a crucial method for patients, HCPs and HCOs, according to them. These findings align with the study (Al‐Maskari & Patterson, 2018), which showed that most HCPs considered EBP a necessary and beneficial method. However, due to a lack of resources in healthcare organizations, nurses, on the other hand, saw EBP as a tough and complex method (Hisham et al., 2016).

The participants reported three EBP barriers in the current study: individual‐, organizational‐ and patient‐related barriers. They declared that the most common organizational barrier is a lack of time to search and find relevant resources and heavy workload related to the nature of the nursing profession and a lack of technical resources. In line with these results, a study done in Saudi Arabia concluded that the three top‐ranked barriers were as follows: the results of the studies are not generalizable to nurses' settings, facilities are inadequate and physicians do not cooperate with the implementation (Alqahtani et al., 2020). Moreover, Anaman‐Torgbor et al. (2022) and Clarke et al. (2021) found that the uptake of EBP among nurses is suboptimal, and nurses experience varying difficulties and barriers to EBP. Conversely, the Egyptian survey, on the other hand, found that lack of time is the least among the top four hurdles faced by the participants. Resistance to change, a lack of access to research information, a lack of authority for implementing study findings and a lack of time were among the obstacles (Baiomy & Khalek, 2015).

Individual barriers to EBP, according to the participants, include a lack of EBP training and staff shortage. These findings were explained by a lack of understanding of the EBP concept and steps because of the limited EBP training programme. These findings agree with earlier research, which found that the majority of barriers among participants were due to a lack of EBP training, which resulted in a lack of EBP knowledge and skills (Clarke et al., 2021). This current study discovered that the participants did not attend any EBP training programmes. This conclusion could be explained by nurses' workload, lack of time and financial resources that hinder them from attending EBP training. This finding is consistent with a study by Alshehri et al. (2017).

In this current study, the age, educational level and work experience of nurses were identified among the individual barriers. Younger, highly educational level and more work‐experienced nurses had a greater understanding, and more willingness to use EBP. Nurses with less experience (fewer years of service) perceive more barriers to the application of EBP than nurses with more experience. Specifically, nurses holding a diploma or post‐graduate degree stated more obstacles than nurses who hold a bachelor's degree. The studies by (Alqahtani et al., 2022; Khoddam et al., 2023; Pitsillidou et al., 2021) are in a similar vein with our study findings.

Regarding patient‐related barriers, the participants indicated that the patients refused to accept any treatment plan that included innovation. Furthermore, patients' families had a strong influence related to the cost of advanced treatment and the inability to pay for the expensive novel therapies. Patients' lack of awareness and engagement in clinical decisions could explain this finding. In addition, the patient's poor overall health makes it difficult to implement a new strategy. This conclusion is in line with a study in which participants stated that the patients were unwilling to adjust their treatment plans or apply EBP (Tacia et al., 2015).

Concerning EBP facilitators, HCOs should give internet access, free time without clinical assignments, leadership support and patient's partnership as reported by the participants. The provision of financial and moral incentives to raise collaboration in EBP can be as well operative in this regard. Similarly, McNett et al. (2022), an inter‐professional team with strong leadership, sufficient resources and stakeholder involvement in a supportive EBP organizational culture, will execute EBP in healthcare settings more successfully. Furthermore, the participants of this study stated that HCOs should increase their competencies by giving EBP educational training. This finding is consistent with Melnyk et al. (2021).

Regarding the feasible method of implementing the EBP strategy, the educational intervention, adopting clinical practice guidelines and clinical protocol were the most prevalent strategies for using and distributing the EBP approach, resulting in more favourable practice environments and reducing the variability of care as reported by the participants, according to the current study. This finding is in line with Khoddam et al. (2023).

## 5 CONCLUSION

According to the findings, it can be concluded that the participants encountered a range of challenges and barriers when implementing EBP in their healthcare context because they lacked the knowledge and abilities necessary to adopt EBP in clinical practice decision‐making. They perceived EBP as a complicated approach. This is because of barriers related to individual, organizational and patient variables. The absence of organizational commitment to implementing EBP led to a lack of support and encouragement. Therefore, it is imperative to encourage the adoption of EBP implementation and provide training on the identified barriers. The results can be used by nurse managers, nurse educators and policymakers at the Ministry of Health to enhance the implementation of EBP.

## 6 RECOMMENDATIONS

The following recommendations are proposed based on the study's findings and conclusion:

1. Enriching the nurses' experiences and skills of the importance of evidence‐based practice.
2. Incorporating evidence‐based practice into workshop‐based staff development programmes and journal clubs to improve knowledge and attitudes towards EBP among the nurses. Besides, EBP mentors would be required to function as EBP facilitators and EBP champions in healthcare.
3. Healthcare organizations should provide a knowledge management system and databases linked to nursing to obtain the information needed for clinical decision‐making as quickly and efficiently as possible.
4. Collaboration between the clinical settings and academic organizations to aid in transferring these novel approaches to the clinical settings.

### 6.1 Limitations and strengths

This study's ability to reflect actual nurses' experiences within the framework of their awareness of the process of creating and implementing EBP is one of its strongest points. These findings, in conjunction with those from related studies, can provide a more thorough understanding of the aspects of implementing evidence in clinical practice and serve as the foundation for the creation of initiatives aimed at extending, preserving and advancing long‐lasting evidence‐based reforms within the healthcare system. This study's qualitative design limits its generalizability to comparable personnel and clinical settings.

## 7 CLINICAL RESOURCES

The key to advancing quality and safety in healthcare [Internet]. Becker's Hospital Review. 2022. Available from: https://www.beckershospitalreview.com/quality/evidence‐based‐practice‐the‐key‐to‐advancing‐quality‐and‐safety‐in‐healthcare.html. The Australian experience of nurses' preparedness for evidence‐based practice. https://onlinelibrary.wiley.com/doi/10.1111/j.1365‐2834.2009.00997.x.

## AUTHOR CONTRIBUTIONS

RAM, WGN and AAA: Made substantial contributions to conception and design, acquisition of data or analysis and interpretation of data. FAA and MA: Involved in drafting the manuscript or revising it critically for important intellectual content. RAM, MA, FAA, WGN and AAA: Given final approval of the version to be published; each author should have participated sufficiently in the work to take public responsibility for appropriate portions of the content. RAM, MA, FAA, WGN and AAA: Agreed to be accountable for all aspects of the work in ensuring that questions related to the accuracy or integrity of any part of the work are appropriately investigated and resolved.

## FUNDING INFORMATION

The University of Bisha through the general research project under grant number UB‐GRP‐19‐1444 funded the study.

## CONFLICT OF INTEREST STATEMENT

The authors declare no conflict of interest.

## ETHICS STATEMENT

Throughout the study, the researchers adhered to the guidelines and ethical standards while working with human respondents required by the Institutional Review Board of the University of Bisha (UBCOM‐RELOC) (H‐06‐BH‐087). Moreover, an official letter from the nursing department of the faculty of applied medical sciences was submitted to the concerned administrative authorities in the selected settings for obtaining authorization and support during the data collection period. This study was performed in accordance with the ethical standards laid down in the 1964 Declaration of Helsinki and its later amendments. The research ethical committee approved the study protocol from the University of Bisha. All study participants gave their informed verbal consent. All study participants were told that all the obtained information they provided would be kept private, used exclusively for research purposes and analysed anonymously. They were also told that they had the right to withdraw from the study for any reason. With concerns about risk, there was not any risk linked to participation. The study findings were shared with the participants for their perspectives and feedback on the appropriateness of the themes. During the data analysis, the participants' names were coded as numbers.

## Data Availability

The datasets generated and analysed during the current study are available upon reasonable request from the corresponding author.
